# Impact of Artificial Intelligence–Generated Content Labels On Perceived Accuracy, Message Credibility, and Sharing Intentions for Misinformation: Web-Based, Randomized, Controlled Experiment

**DOI:** 10.2196/60024

**Published:** 2024-12-24

**Authors:** Fan Li, Ya Yang

**Affiliations:** 1 School of Journalism and Communication Beijing Normal University Beijing China

**Keywords:** generative AI, artificial intelligence, ChatGPT, AIGC label, misinformation, perceived accuracy, message credibility, sharing intention, social media, health information

## Abstract

**Background:**

The proliferation of generative artificial intelligence (AI), such as ChatGPT, has added complexity and richness to the virtual environment by increasing the presence of AI-generated content (AIGC). Although social media platforms such as TikTok have begun labeling AIGC to facilitate the ability for users to distinguish it from human-generated content, little research has been performed to examine the effect of these AIGC labels.

**Objective:**

This study investigated the impact of AIGC labels on perceived accuracy, message credibility, and sharing intention for misinformation through a web-based experimental design, aiming to refine the strategic application of AIGC labels.

**Methods:**

The study conducted a 2×2×2 mixed experimental design, using the AIGC labels (presence vs absence) as the between-subjects factor and information type (accurate vs inaccurate) and content category (for-profit vs not-for-profit) as within-subjects factors. Participants, recruited via the Credamo platform, were randomly assigned to either an experimental group (with labels) or a control group (without labels). Each participant evaluated 4 sets of content, providing feedback on perceived accuracy, message credibility, and sharing intention for misinformation. Statistical analyses were performed using SPSS version 29 and included repeated-measures ANOVA and simple effects analysis, with significance set at *P*<.05.

**Results:**

As of April 2024, this study recruited a total of 957 participants, and after screening, 400 participants each were allocated to the experimental and control groups. The main effects of AIGC labels were not significant for perceived accuracy, message credibility, or sharing intention. However, the main effects of information type were significant for all 3 dependent variables (*P*<.001), as were the effects of content category (*P*<.001). There were significant differences in interaction effects among the 3 variables. For perceived accuracy, the interaction between information type and content category was significant (*P*=.005). For message credibility, the interaction between information type and content category was significant (*P*<.001). Regarding sharing intention, both the interaction between information type and content category (*P*<.001) and the interaction between information type and AIGC labels (*P*=.008) were significant.

**Conclusions:**

This study found that AIGC labels minimally affect perceived accuracy, message credibility, or sharing intention but help distinguish AIGC from human-generated content. The labels do not negatively impact users’ perceptions of platform content, indicating their potential for fact-checking and governance. However, AIGC labeling applications should vary by information type; they can slightly enhance sharing intention and perceived accuracy for misinformation. This highlights the need for more nuanced strategies for AIGC labels, necessitating further research.

## Introduction

### Background

The dissemination of disordered information on social media has long been a critical area of study, significantly impacting public interest [1]. With rapid advancements in technologies such as deepfakes [2] and generative artificial intelligence (AI) [3,4], individuals now have various tools, sources, and channels at their disposal to create misinformation or even false information. Although technological developments offer convenience [5], they also inevitably complicate the fact-checking and governance of misinformation on social media. In particular, in the era of generative AI, such technologies can become crucial for producing misinformation, either actively or passively [6], including news production [7] and health care provision [6]. Research observed that, even before the advent of ChatGPT (based on Generative Pre-trained Transformer [GPT]-3.5), humans struggled to distinguish between AI-generated content (AIGC) and human-generated content (HGC) during the GPT-2 era. This is particularly concerning for medical information that requires rigorous verification, as it can have a significant impact on health care and the general public [8]. Although generative AI can assist with writing summaries, the credibility and accuracy of AIGC are not guaranteed to be 100% [9,10]. This ongoing issue underscores the challenge of effectively governing misinformation as AI capabilities continue to advance [7].

AI is iterating at an unimaginable pace, leading to an even more challenging distinction between AIGC and HGC for the average internet user in the future. What is more important, as AIGC increasingly becomes part of social media content, driven by online traffic and profit motives, the presence of misinformation will be inevitable. Therefore, it is essential for platforms to implement governance measures to help users differentiate between AIGC and HGC. Previous research [11] has proposed nudging and boosting as 2 types of interventions, with nudging involving the integration of cognitive cues into interface design through proactive notifications. Current popular measures like accuracy prompts [12] and warning labels [13] fall under the category of nudging interventions. Research has shown that nudging can effectively reduce the spread of misinformation [13]. However, the effectiveness of nudging interventions can be influenced by factors such as political affiliation [7] and geographical area (urban, suburban, rural) [14]. Further, the effects of nudging interventions might also lead to divergent attitudes and unintended consequences [15], such as the implied truth effect, where the absence of interventions could imply the veracity of information [16], illustrating the complexity of implementing such measures. Clearly, the factors affecting nudging and the additional impacts caused by nudging are extensive and multifaceted.

Facing the rise in generative AI, in 2023, the Chinese social media platform Douyin (Chinese version of TikTok) issued a platform operation policy requiring content creators to prominently label AIGC [17], with similar initiatives seen on other platforms like Zhihu and Little Redbook (Xiaohongshu). These AIGC labels, akin to accuracy prompts [12], serve as a nudging intervention. Despite the widespread adoption of these labels, research on the factors that influence their effectiveness and the consequences they yield remains sparse. Previous studies have uniquely combined electroencephalogram technology with behavioral experiments to explore how AIGC labels impact users’ perceptions of automated news, including their effect on content credibility and whether the news type interacts with these labels, alongside cognitive-physiological impacts [18], indicating that AIGC labels prompt users to engage in deeper information processing, consequently lowering the perceived credibility of the content. This suggests that, although AIGC labels are intended to enhance transparency and reliability, they may paradoxically lead users to view labeled content with increased skepticism, highlighting a complex dynamic between labels’ presence and users’ perceptions [18].

AIGC labels do not exist in isolation; they may be influenced by various contextual factors. It is necessary to integrate AIGC labels with the surrounding context, such as combining them with images, videos, advertisements, and other information. AIGC labels may interact with these factors as described in the following paragraphs [19].

First, regarding information type and AIGC labels, the governance of misinformation or inaccurate information has been a crucial focus of academic research. The regulation of misinformation dissemination when AI is involved has become a new issue. Previous studies have found an interaction effect between accuracy prompts and information type, known as the “implied truth” effect, where people may mistakenly perceive unlabeled misinformation as more credible [16]. This finding has also been supported in research on labels related with stance and credibility, which may inadvertently promote the spread of misinformation [20]. Thus, there can be an interaction effect between inaccurate information and AIGC labels, as well as between accurate information and AIGC labels. In summary, there could be a potential interaction effect between information type (accurate and inaccurate information) and AIGC labels.

Second, in terms of content category and AIGC labels, the effectiveness of AIGC labels may vary depending on the content category, demonstrated by the presence of cases in which AIGC labels are less effective for certain types of content [13]. Previous research on labels has typically examined them in isolation [21], but some researchers have begun studying the interaction between labels and information types from a contextual perspective [22]. Though there is substantial research on nudging interventions in specific content areas, such as political content [13], climate change [23], and pandemic-related information [24], little research has explored the relationship between nudging interventions and content category. Based on the profitability of the content, online content can be discretely divided into for-profit and not-for-profit categories, which is a common classification method [25]. This aligns with the current landscape of social media, which is filled with for-profit content, such as advertisements, and not-for-profit content, such as news. From a content category perspective, the aforementioned studies mainly focus on not-for-profit content. However, for-profit content may also influence the effectiveness of AIGC labels. Additionally, different topics within the same content category may produce unexpected effects [26].

In addition, the impacts of AIGC labels have extended beyond awareness levels. According to recent studies, such nudging interventions not only influence message credibility [18,23] or perceived accuracy, which is the capability to differentiate between accurate and inaccurate information to some extent [26], but also affect sharing intention [27]. This phenomenon can be attributed to a psychological inoculation effect induced by AIGC labels [28], which acts as a preemptive defense against misinformation, giving users a latent resistance before they encounter false information. Therefore, by preemptively introducing users to the concept of AIGC, these labels do impact message credibility, sharing intention, and even the capability to identify misinformation.

### Objective

AIGC has visibly altered the content production and dissemination ecosystem on social media. In practical applications, AIGC labels have become cognitive cues on some social media platforms to help users distinguish between AIGC and HGC, functioning through nudging interventions. However, theoretical and empirical research related to AIGC labels is significantly lacking, and extensive research around AIGC labeling is required. Therefore, this study aimed to investigate AIGC labels to guide practical application. This research focused on addressing 2 main issues: (1) the predictive factors that influence the effectiveness of AIGC labels as a nudging intervention and (2) the impacts that these AIGC labels have on social media users. This study used a web-based experimental method. The independent variables were the AIGC labels (presence vs absence), information type (accurate vs inaccurate), and content category (for-profit vs not-for-profit). The dependent variables were perceived accuracy, message credibility, and sharing intention.

This study aimed to address the following research questions:

- RQ1: Will AIGC with labels impact (1) perceived accuracy, (2) message credibility, and (3) sharing intention about misinformation?
- RQ2: Are the effects of AIGC labels on perceived accuracy, message credibility, and sharing intention influenced by the (1) information type and (2) content category?

## Methods

### Design

This study examined 3 independent variables, with 1 between-subjects variable, namely the AIGC labels (presence vs absence), and 2 within-subjects variables, namely the information type (accurate vs inaccurate) and content category (for-profit vs not-for-profit). Based on this, a 2×2×2 mixed experimental design with 2 groups—an experimental group with AIGC labels and a control group without AIGC labels—was used. This study was not a clinical trial to recruit participants but a web-based experiment to measure the effect of internet governance measures, so clinical trial registration was not required. Participants in both the experimental and control groups were exposed to 4 combinations of information type and content category, resulting in a total of 8 experimental conditions. Hence, this study used a 2×2×2 mixed experimental design, as outlined in Table 1.

**Table 1:** Overview of the experimental design, which used 3 independent variables at 2 levels each and 8 experimental conditions in total.

Information type	With AIGC^a^ labels (A)		Without AIGC labels (B)	
	For-profit (1)	Not-for-profit (2)	For-profit (1)	Not-for-profit (2)
Accuracy (a)	Aa1	Aa2	Ba1	Ba2
Inaccuracy (b)	Ab1	Ab2	Bb1	Bb2

^a^AIGC: artificial intelligence–generated content.

### Participants

This study recruited participants through the data platform Credamo [29], which boasts a representative sample size of over 3 million people from China and has been used by multiple universities and research institutions [30-32], with publications in top-tier journals across various fields. There were no specific demographic quota requirements for the participants in this study. On the Credamo data platform, 957 participants were recruited, with 476 in the group with AIGC labels and 481 in the group without AIGC labels. The data for this study were collected in April 2024. The participants agreed to participate and received a small compensation for their participation.

To ensure the data quality of the online experiment, this study recruited 7 participants to take part in an offline behavioral experiment before the formal experiment. The participants were instructed to complete the task with both attention and speed, resulting in a minimum time threshold of 151.8 seconds to complete the experiment (Table 2). Thus, participants in the formal online experiment who completed the survey in less than 151.8 seconds were excluded from the analysis, as this was deemed insufficient to fully engage with the experimental content. A total of 157 invalid responses were removed. The final valid sample consisted of 800 participants, with 400 in the group with labels and 400 in the group without labels. All participants were Chinese (254 women and 546 men), and the majority had at least an undergraduate-level education (736 individuals).

**Table 2:** The minimum time taken to complete the task by 7 participants in an offline behavioral experiment.

Participants	Time (seconds)
HZH	155
WSN	157
YSJ	146
JBX	163
ZYX	145
LZX	149
SHC	148

### Procedures

#### Overview

For this study, participants were recruited through the Credamo platform. Since the research involved a mixed design with between-subjects variables, to prevent participants from participating in both groups in the experiment, recruitment for the group with AIGC labels was initiated first. Upon completion of recruitment for the group with AIGC labels, leveraging the sophisticated distribution mechanism of the recruiting platform, those who had already participated in the group with AIGC labels were excluded, and recruitment for the group without AIGC labels commenced.

**Figure 1:**
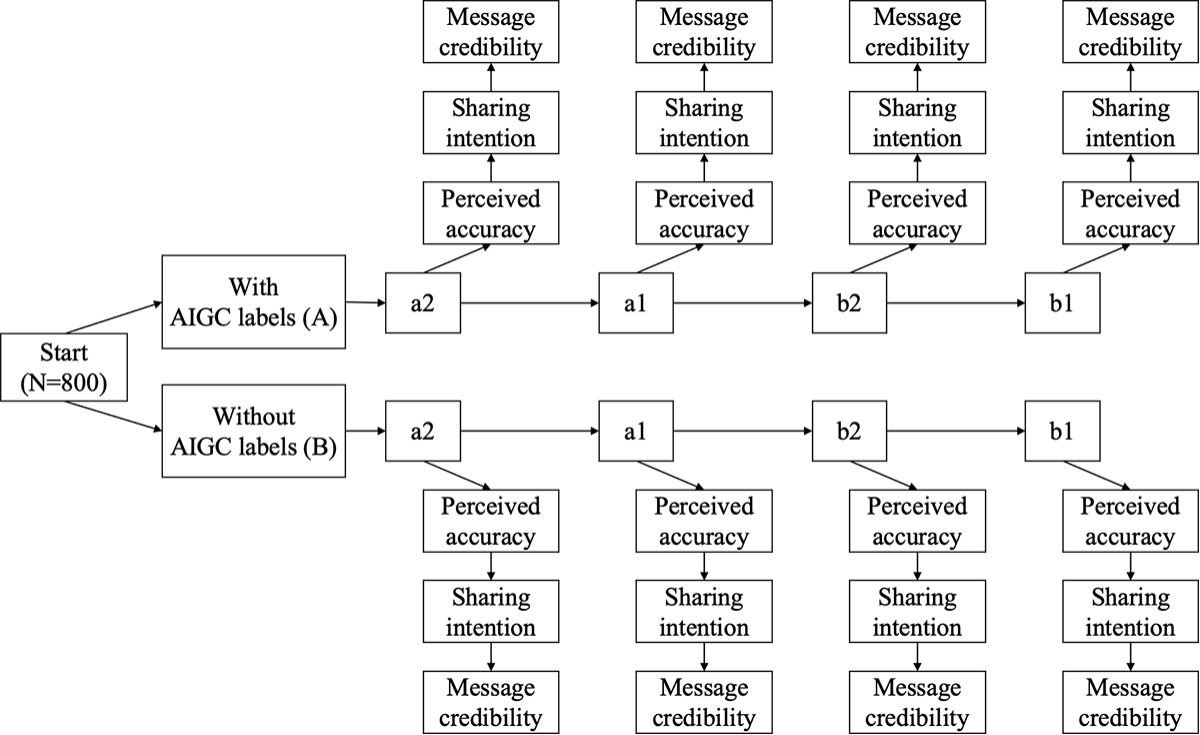
Overview of the 3-factor mixed experimental design in a randomized controlled trial involving 800 participants. AIGC: artificial intelligence–generated content.

Once participants entered the experiment, they began by signing an informed consent form. They were then presented with 4 types of content: accurate and not-for-profit (a2), accurate and for-profit (a1), inaccurate and not-for-profit (b2), and inaccurate and for-profit (b1). Notably, participants were not informed whether the information was accurate. After reading each piece of content carefully, the perceived accuracy, the message credibility, and their sharing intention were measured using scales. This process was repeated 4 times. After completing the demographic survey, participants submitted their responses. The experimental procedures are shown in Figure 1.

#### Materials

Regarding the AIGC labels, in this study, a key focus was on the form and position of AIGC labels. The current forms of AIGC labels on Chinese social media platforms like Douyin and Little Redbook include "Content Suspected to be AI-Generated, Please Discern Carefully,” “Author’s Declaration: Content Generated by AI,” and “Suspected to Contain AI-Created Information, Please Check for Authenticity.” We categorized existing AIGC labels as either prompts regarding the accuracy of AI generation or as authors’ declarations of AIGC. Distinct from prompts about accuracy, which remind users to prudently discern content, AIGC labels primarily inform users that the content was generated with the assistance of AI; they serve as a nudge, providing information to users in a purely informative manner. Therefore, this study approached the use of AIGC labels from the perspective of restructuring the online environment. It used labels to notify users of AIGC involvement without necessitating a declaration by the author. In accordance with government regulations on the operation of generative AI on social media platforms, content labeling methods are categorized into explicit and implicit watermark labels. The content should contain information such as “Generated by Artificial Intelligence,” which is recommended to be placed at the corners of the screen, occupying no less than 0.3% of the screen area or with a text height not less than 20 pixels [33]. Aligning with the AIGC labels currently used on social media platforms, the AIGC labels in this study read as "[Warning Signal in Yellow] Content generated by AI” and were located on the lower left of the AIGC.

Regarding the information types, this study primarily investigated the effects of AIGC labels and the factors that influence the efficacy of these labels. As AIGC labels normally appear within the body of text, this study focused not only on the headlines but also on the main content. AIGC labels do not function in isolation during the dissemination process and can be affected by various factors. The type of information can exert an influence [5,34]. When generative AI is used as a legitimate auxiliary tool, content produced with the assistance of generative AI, after passing through a vetting process, constitutes accurate information. Conversely, when generative AI is maliciously used, it may be involved in the production of misleading or false information.

Therefore, in this study, the type of information was categorized as either accurate or inaccurate. The experimental materials underwent a rigorous screening and evaluation process. The accurate information used in the study was real content circulated on social media, with clearly identified authors, channels, and sources. The inaccurate information was generated by AI and contained objectively false content. Combining the 2 independent variables—information type and content category—at 2 levels, there were 4 groups of experimental materials. Initially, 8 materials were collected for each group, totaling 32 materials.

First, to ensure the generalizability of the materials, a nonspecialist scholar with a PhD was invited to conduct the first review. Materials that included data descriptions were removed, leaving 6 materials per group after the initial screening, for a total of 24 materials. Second, to ensure that the experimental materials did not produce significant differences in the dependent variables (perceived accuracy, message credibility, and sharing intention), a pilot study was conducted with 50 participants recruited via the Credamo platform (19 men, 31 women). A 1-way ANOVA was used to exclude materials that showed significant differences, and based on pairwise comparisons and mean plots, the materials with the most similar results were selected. After this process, each group was left with 3 materials, totaling 12 materials, as shown in Table 3.

**Table 3:** Evaluated results of pilot testing with the experimental materials with 50 participants.

Information type and content category		Number assigned to the experimental materials during the pretest	Perceived accuracy, mean (SD)	Message credibility, mean (SD)	Sharing intention, mean (SD)
**Accurate**					
	**Not-for-profit**				
		4	5.42 (1.144)	5.67 (1.011)	5.04 (1.577)
		5	5.6 (1.245)	5.65 (1.045)	5.12 (1.272)
		6	5.38 (1.441)	5.38 (1.234)	4.88 (1.780)
	**For-profit**				
		10	4.54 (1.606)	4.63 (1.523)	3.94 (1.845)
		11	4.94 (1.168)	5.07 (1.175)	4.28 (1.604)
		12	4.38 (1.760)	4.61 (1.713)	4.34 (1.825)
**Inaccurate**					
	**Not-for-profit**				
		18	3.26 (1.536)	3.15 (1.608)	3 (1.829)
		21	3.36 (1.687)	3.23 (1.662)	3.02 (1.708)
		24	3.24 (1.636)	3.23 (1.594)	2.9 (1.787)
	**For-profit**				
		27	2.52 (1.266)	2.43 (1.203)	1.98 (1.301)
		28	2.68 (1.253)	2.47 (1.212)	2.14 (1.400)
		31	2.6 (1.414)	2.48 (1.318)	2.08 (1.496)

Regarding the content categories, the impact of information types is contingent on specific content categories, with for-profit versus not-for-profit being a common criterion for differentiation [35,36]. This dichotomy allows for the classification of content into for-profit and not-for-profit categories. Here, not-for-profit content was represented by news articles, while for-profit content was represented by advertisements.

To minimize the influence of varying themes within the experimental materials [26], this study selected health-related content for both the for-profit and not-for-profit categories. Since the onset of the pandemic, health-themed news has increasingly captured the attention of the general populace, with inaccurate health information potentially having direct consequences on physical and psychological well-being [37]. In addition, food safety issues are closely linked to consumer health, with a high demand for information related to food safety [38] and a practical problem faced by China and globally [39]. For instance, social media or influencer-recommended foods may not meet the nutritional requirements of the human body [40], and sponsorship from unhealthy food advertisements, such as those for alcohol and sugary foods, is prevalent [41].

To ensure the experimental validity, it was crucial to maintain a consistent text length across experimental materials, thus avoiding any unintended effects caused by word count disparities [42]. In this study, we first gathered accurate information in real practice and used the text length of accurate information as a basis. On this foundation, generative AI, such as ChatGPT, was used to produce the misinformation, which then was reviewed and vetted by experts.

In this study, news information was selected to represent not-for-profit content, with accurate not-for-profit content sourced from the official health website of Health Key News Section on China Central Television [43]. To eliminate the influence of other informational cues, details such as dates, reporter names, newspaper names, and layout information were omitted from the specific news content. Accurate for-profit content was represented by advertisements from the rice cultural and creative brand Zhangshengguli posted on social media. Descriptive statistics of the accurate information revealed that the average word count for each piece of experimental material is 273 words. Although the for-profit content had more words than the not-for-profit content, the difference in reading speed was much greater than this discrepancy [44], so the differences caused by the mean and standard deviation are negligible. Building on this, we applied ChatGPT for the generation of misinformation, overseen and vetted by experts, setting prompt words based on the word count and standard deviation of the accurate information. Descriptive statistics regarding the word count of the 4 types of experimental materials are detailed in Table 4.

**Table 4:** Descriptive statistics of the word count for the experimental materials (overall: mean 268, SD 13.638 words).

Information type and content category		Word count, n	Word count, mean (SD)
**Accurate (a)**			273 (10.607)
	Not-for-profit (2)	265	
	For-profit (1)	280	
**Inaccurate (b)**			264 (19.092)
	Not-for-profit (2)	277	
	For-profit (1)	250	

**Figure 2:**
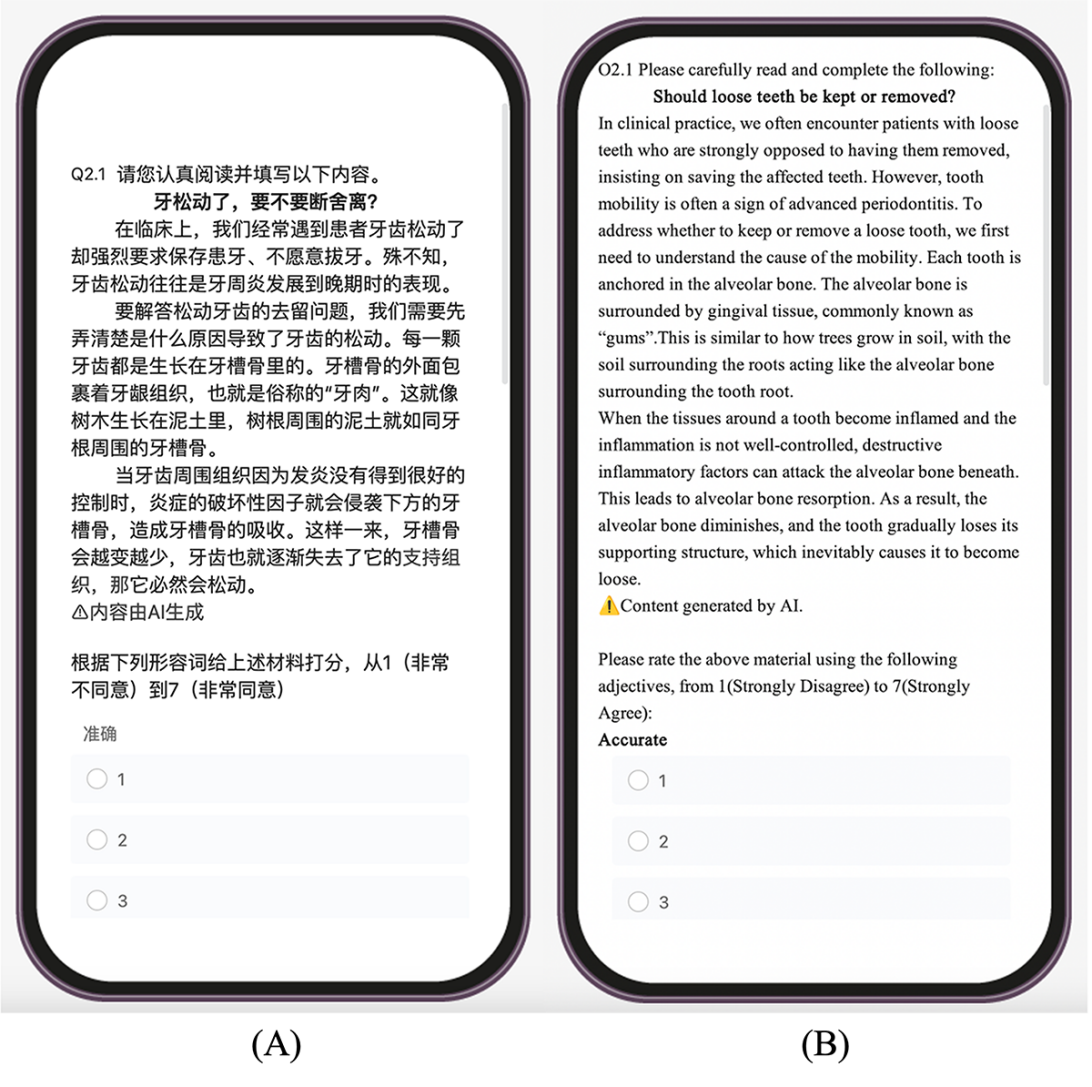
Presentation of the experiment materials in (A) Chinese and (B) English.

There were a few differences in the word count between the accurate and inaccurate information, but these differences were considered negligible due to the human reading speed being far greater than the variance [44]. The presentation of the experimental materials is shown in Figure 2.

### Outcome Measures

#### Perceived Accuracy

After participants viewed each experimental material, their perceived accuracy of the material was assessed using an item from the Information Credibility Scale [45], in which the “accurate” item was outlined by Luo et al [26].

#### Message Credibility

The Information Credibility Scale [45] has demonstrated strong validity and internal consistency, evidenced by a relatively high scale reliability (Cronbach =.87) [45]. Subsequently, this scale has been widely adopted and validated in numerous studies [23,26]. In this study, the scale was adapted and translated to include 3 items that assess accuracy, authenticity, and believability. These items were rated on a 7-point Likert scale, ranging from 1 (strongly disagree) to 7 (strongly agree), to gauge credibility of the information presented. The sum of these scores formed an overall credibility score, which can range from 3 to 21, where higher scores indicate greater message credibility. In our sample, the scale demonstrated excellent internal consistency (Cronbach =.941).

#### Sharing Intention

This study adapted a measurement approach originally developed by Pennycook et al [46] to assess participants’ intentions about information sharing. After viewing each piece of experimental material, participants were posed a question to capture their likelihood of sharing the content on social media platforms, such as WeChat, QQ, Little Redbook, and Douyin. The question was “Would you consider sharing this message on social media?”, and responses were recorded on a 7-point Likert scale, where 1 represents “strongly disagree” and 7 represents “strongly agree.”

### Demographic Questions

At the conclusion of the experiment, participants were asked to provide demographic information including their gender (male, female), age (<18, 18-25, 26-29, 30 years), exact date of birth (by selecting a specific date), level of education (high school or below, undergraduate, master graduate, doctorate or above), and primary city of residence (by selecting both the provincial and municipal area).

### Statistical Analysis

Descriptive analyses were first conducted to examine demographic characteristics and outcome data. The influence of 3 variables—AIGC labels (presence vs absence), information type (accurate vs inaccurate), and content category (for-profit vs not-for-profit)—on perceived accuracy, message credibility, and information sharing intention was then assessed using repeated measures ANOVA in SPSS version 29 (IBM Corp). When significant interaction effects were identified, simple effect analyses were performed to explore specific differences among the conditions.

An a priori power analysis was conducted using G*Power software [47]. Based on an assumed medium effect size (*f*= 0.25), at least 36 participants were needed to achieve adequate power, with a minimum of 18 participants per experimental group, using a significance threshold of α=.05 and aiming for a power of 95%. Statistical analysis was performed using SPSS version 29 [48]. To accommodate the unpredictability associated with mobile experiments, the sample size was increased to 800 participants.

### Ethical Considerations

On April 22, 2024, this study obtained ethical approval from the ethics committee of the School of Journalism and Communication, Beijing Normal University (approval number BNUJ&C20240422002). The research commenced after obtaining informed consent from all participants, who were informed that their data would be used anonymously and that they could withdraw from the study at any time without penalty. Throughout the study, all data were collected and reported anonymously to ensure participant confidentiality, and no identifiable personal data were included. Upon completing their participation, individuals received a small remuneration of ¥1 (US $0.14) through the Credamo platform.

## Results

### Sample Characteristics

By April 2024, this study recruited 957 participants. Of these, 157 participants (16.4% of the initial sample) were excluded due to their extremely short completion times, suggesting that they did not thoroughly engage with the experimental materials. Consequently, the final valid sample comprised 800 participants, randomly divided between 2 groups: 400 in the group with AIGC labels and 400 in the group without AIGC labels. The demographic data are shown in Table 5.

**Table 5:** Participant demographic data (N=800).

Characteristics			Results, n (%)
**Gender**			
	Male	254 (31.8)	
	Female	546 (68.2)	
**Age (years)**			
	<18	1 (0.1)	
	18-25	193 (24.1)	
	26-29	135 (16.9)	
	≥30	471 (58.9)	
**Education level, n (%)**			
	High school and below	64 (8)	
	Bachelor’s degree	593 (74.1)	
	Master’s degree	127 (15.89)	
	Doctorate and above	16 (2)	

### Primary Outcomes

#### Perceived Accuracy

The main effect of AIGC labels was not significant. However, the main effect of information type was significant (*F*_1, 798_=498.803, *P<*.001, η_p_^2^=0.385), as was the main effect of content category (*F*_1, 798_=367.142, *P*<.001, η_p_^2^=0.315). Following the standards by Cohen [49], both the information type and content category significantly influenced perceived accuracy when acting independently, and they substantially explained the variance in perceived accuracy. The interaction between information type and content category was also significant (*F*_1, 798_=7.835, *P*=.005, η_p_^2^=0.01), indicating that the combination of information type and content category had a significant, albeit small, explanatory power effect on the dependent variable. Other interactions were not significant as shown in Table 6 and Table 7.

**Table 6:** Main effects, interaction effects, and pairwise comparison statistics for the perceived accuracy of artificial intelligence–generated content (AIGC).

Information type, content category, and AIGC labels				Rating, mean (SD)^a^
**Accurate**				
	**For-profit**			
		AIGC labels present	4.940 (1.424)	
		AIGC labels absent	4.978 (1.494)	
	**Not-for-profit**			
		AIGC labels present	5.673 (1.113)	
		AIGC labels absent	5.680 (1.205)	
**Inaccurate**				
	**For-profit**			
		AIGC labels present	3.718 (1.649)	
		AIGC labels absent	3.695 (1.782)	
	**Not-for-profit**			
		AIGC labels present	4.640 (1.556)	
		AIGC labels absent	4.638 (1.635)	

^a^Rated on a 7-point Likert scale, ranging from 1 (strongly disagree) to 7 (strongly agree).

**Table 7:** Main effects, interaction effects, and pairwise comparison statistics for the perceived accuracy of artificial intelligence–generated content (AIGC).

Conditions	*F* (*df*)	*P* value	η_p_^2^	Pairwise comparisons
AIGC labels	0.005 (1, 798)	.95	0	—^a^
Information type	498.803 (1, 798)	<.001	0.385	Accurate>inaccurate
Content category	367.142 (1, 798)	<.001	0.315	Not-for-profit>for-profit
Information type × AIGC labels	0.117 (1, 798)	.73	0	—
Content category × AIGC labels	0.003 (1, 798)	.95	0	—
Information type × content category	7.835 (1, 798)	.005	0.01	Accurate>inaccurate; not-for-profit>for-profit
Information type × content category × AIGC labels	0.106 (1, 798)	.75	0	—

^a^Not applicable.

**Figure 3:**
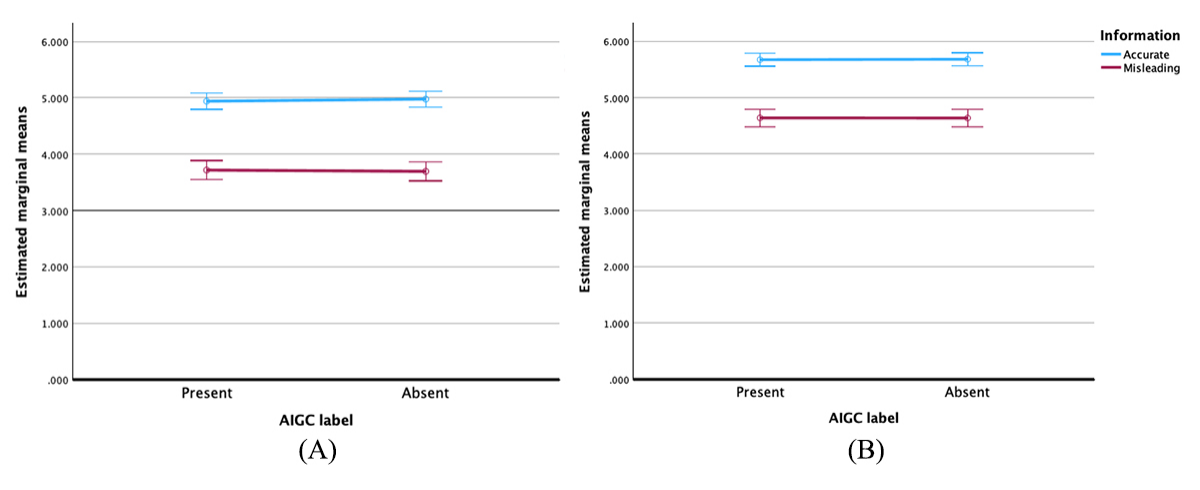
Error bars and 95% CIs of the values for perceived accuracy of accurate and misleading artificial intelligence–generated content (AIGC) for (A) for-profit content and (B) not-for-profit content.

As indicated in Figure 3, when the information type was accurate, the presence of AIGC labels slightly reduced the perceived accuracy regardless of content type; however, this effect was not statistically significant. Conversely, when the information type was inaccurate, AIGC labels slightly enhanced the perceived accuracy irrespective of the content type, though this lacked statistical significance.

#### Message Credibility

As shown in Table 8 and Table 9, the main effect of AIGC labels was not statistically significant. However, significant main effects were observed for information type (*F*_1, 798_=668.596, *P*<.001, η_p_^2^=0.456) and content category (*F*_1, 798_=442.506, *P*<.001, η_p_^2^=0.357). According to the standards set by Cohen [49], different information types and content categories significantly influenced message credibility when acting independently, accounting for a substantial proportion of the variance in credibility. Additionally, the interaction between information type and content category was significant (*F*_1, 798_=18.37, *P*<.001, η_p_^2^=0.023), indicating that their combination had a significant, albeit small, effect on the dependent variable. Other interactions were not significant.

**Table 8:** Statistical data for message credibility of artificial intelligence–generated content (AIGC).

Information type, content category, and AIGC labels				Rating, mean (SD)^a^
**Accurate**				
	**For-profit**			
		AIGC labels present	4.952 (1.340)	
		AIGC labels absent	5.045 (1.425)	
	**Not-for-profit**			
		AIGC labels present	5.687 (1.013)	
		AIGC labels absent	5.761 (1.115)	
**Inaccurate**				
	**For-profit**			
		AIGC labels present	3.520 (1.648)	
		AIGC labels present	3.571 (1.742)	
	**Not-for-profit**			
		AIGC labels present	4.586 (1.557)	
		AIGC labels present	4.560 (1.600)	

^a^Rated on a 7-point Likert scale, ranging from 1 (strongly disagree) to 7 (strongly agree).

**Table 9:** Main effects, interaction effects, and pairwise comparison statistics for the message credibility of artificial intelligence–generated content (AIGC).

Conditions	*F* (*df*)	*P* value	η_p_^2^	Pairwise comparisons
AIGC labels	0.462 (1, 798)	.50	0.001	—^a^
Information type	668.596 (1, 798)	<.001	0.456	Accurate>inaccurate
Content category	442.506 (1, 798)	<.001	0.357	Not-for-profit>for-profit
Information type * AIGC labels	0.501 (1, 798)	.48	0.001	—
Content category × AIGC labels	0.331 (1, 798)	.57	0	—
Information type × content category	18.37 (1, 798)	<.001	0.023	Accurate>inaccurate; not-for-profit>for-profit
Information type × content category × AIGC labels	0.166 (1, 798)	.68	0	—

^a^Not applicable.

**Figure 4:**
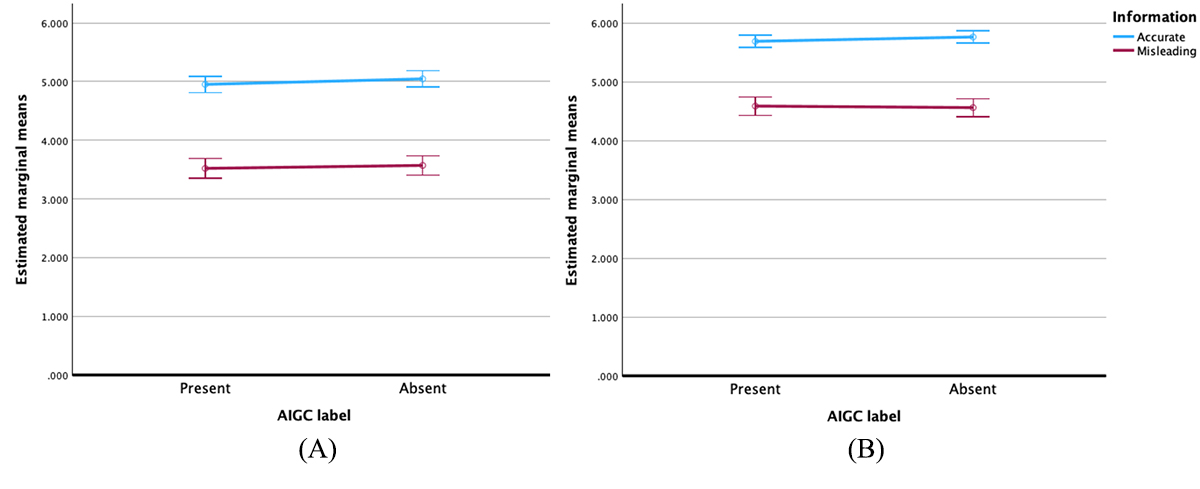
Error bars and 95% CIs of the values for the message credibility of accurate and misleading artificial intelligence–generated content (AIGC) for (A) for-profit content and (B) not-for-profit content.

Figure 4 illustrates that, for for-profit content, AIGC labels marginally reduced the perceived credibility of both accurate and inaccurate information; however, these effects were not statistically significant. Conversely, in the context of not-for-profit content, AIGC labels slightly decreased the message credibility for accurate information. Interestingly, for inaccurate information within not-for-profit content, AIGC labels appeared to slightly enhance message credibility, though this effect also lacked statistical significance.

#### Information Sharing Intention

As seen in Table 10 and Table 11, the main effect of AIGC labels was not significant. The main effect of information type was significant (*F*_1, 798_=496.406, *P*<.001, η_p_^2^=0.384), and the main effect of content category was significant (*F*_1, 798_=387.801, *P*<.001, η_p_^2^=0.327), indicating that different information types and content categories individually had a significant impact on message credibility and explained a large portion of the variance in credibility [49]. The interaction between information type and AIGC labels was significant (*F*_1, 798_=7.158, *P*=.008, η_p_^2^=0.009), suggesting that the combination of information type and AIGC labels had a significant but very small effect on the dependent variable. The interaction between information type and content category was also significant (*F*_1, 798_=37.388, *P*<.001, η_p_^2^=0.045), indicating that this combination had a significant and modest effect on the dependent variable. Other interactions were not significant.

**Table 10:** Statistical data for the sharing intention of artificial intelligence–generated content (AIGC).

Information type, content category, and AIGC labels				Rating, mean (SD)^a^
**Accurate**				
	**For-profit**			
		AIGC labels present	4.540 (1.699)	
		AIGC labels absent	4.738 (1.768)	
	**Not-for-profit**			
		AIGC labels present	5.225 (1.354)	
		AIGC labels absent	5.393 (1.295)	
**Inaccurate**				
	**For-profit**			
		AIGC labels present	3.190 (1.845)	
		AIGC labels absent	3.208 (1.963)	
	**Not-for-profit**			
		AIGC labels present	4.478 (1.893)	
		AIGC labels absent	4.253 (1.867)	

^a^Rated on a 7-point Likert scale, ranging from 1 (strongly disagree) to 7 (strongly agree).

**Table 11:** Main effects, interaction effects, and pairwise comparison statistics for the sharing intention of artificial intelligence–generated content (AIGC).

Conditions	*F* (*df*)	*P* value	η_p_^2^	Pairwise comparisons
AIGC labels	0.189 (1, 798)	.66	0	—^a^
Information type	496.406 (1, 798)	<.001	0.384	Accurate>inaccurate
Content category	387.801 (1, 798)	<.001	0.327	Not-for-profit>for-profit
Content category × AIGC labels	7.158 (1, 798)	.008	0.009	Accurate>inaccurate
Information type × content category × AIGC labels	2.135 (1, 798)	.14	0.003	—
Information type	37.388 (1, 798)	<.001	0.045	Accurate>inaccurate; not-for-profit>for-profit
Information type × AIGC labels	1.714 (1, 798)	.19	0.002	—

^a^Not applicable.

**Figure 5:**
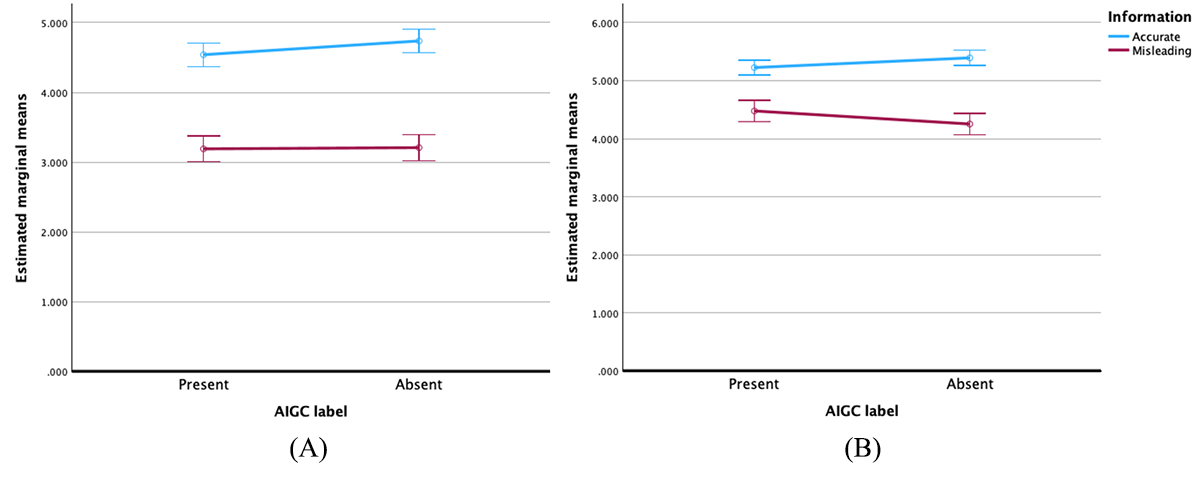
Error bars and 95% CIs of the values for sharing intention of accurate and misleading artificial intelligence–generated content (AIGC) for (A) for-profit content and (B) not-for-profit content.

Figure 5 illustrates that, when the content category was for-profit, AIGC labels slightly decreased sharing intention about accurate information and marginally reduced sharing intention about inaccurate information, though these effects were not statistically significant. When the content type was not-for-profit, AIGC labels also slightly decreased sharing intention about accurate information. However, it was noteworthy that AIGC labels increased sharing intention about inaccurate information to a certain degree, though this was not statistically significant.

## Discussion

### Principal Findings

This study aimed to assess the impact of a new internet governance initiative, AIGC labels, and explore factors that might influence their effectiveness. The findings revealed that AIGC labels do not significantly affect perceived accuracy, message credibility, or sharing intention of users. AIGC labels function as a nudging intervention, helping users distinguish between AIGC and HGC. However, AIGC labels serve an informative role [11], intending to alter cognition rather than actively guide users’ behaviors, which may explain why they have limited influence on users’ perceptions. Importantly, they do not worsen users’ views of platform content or the platform itself, indicating that AIGC labels are a viable strategy for fact-checking and internet governance.

On a physiological level, cognitive neuroscience explains the underlying mechanism of the informative function of AIGC labels, which increase users’ attention to the content they read and deepen cognitive processing, leading to more cautious judgments [18]. This has been corroborated by other studies, which found that labels prompting users to recognize content from minority or anonymous sources encourage more careful consideration of such information [50]. This study highlighted the potential of AIGC labels to foster more cautious content evaluation, contributing to the adaptive development of a nudging [11] approach in internet governance in the generative AI era.

In fact, AIGC labels also play a crucial role in alignment. Current research on AI alignment focuses primarily on value and moral alignment [51-53], but alignment is a complex system-level issue that should not be limited to data and code between humans and AI. Focusing solely on techniques to learn from feedback and handle distribution shifts [54] is a relatively narrow approach. More importantly, we need to address aspects that have been previously overlooked—namely, exploring alignment from the perspective of human-computer interaction and interface design [55]. AIGC labels, by reshaping the interface of the virtual ecosystem, add nudging value, helping users align AIGC with HGC. Addressing this issue requires collaboration between governments, social media platforms, and content creators and producers to label AIGC. This can restructure the online environment without compromising users’ choices, using subtle symbolic cues to prompt users [11] and influence their cognition and behavior. Overall, AIGC labels can enrich the theoretical framework of AI alignment, suggesting new directions for enhancing the integration of AI technology into social platforms.

In addition, AIGC labels do not function in isolation, instead interacting with both the information type and content category to create a comprehensive effect. This study examined how AIGC labels influence perceived accuracy, message credibility, and sharing intention across both accurate and inaccurate information and for both for-profit and not-for-profit content. Notably, our findings indicated that both the information type and content category significantly affect these perceptions, and in particular, accurate information and not-for-profit content were perceived more favorably than their counterparts. Although there is extensive research [56,57] highlighting a general distrust and negative attitude toward for-profit content [58], our results revealed a significant interaction between information type and content category.

This study focused on the specific impact of AIGC labels under various informational conditions. We found that, for accurate information, whether for-profit or not-for-profit content, the presence of an AIGC label slightly reduced perceived accuracy, message credibility, and sharing intention, though these effects were not statistically significant. This suggests that, although AIGC labels do influence users’ perceptions, their impact is relatively modest and manageable. For inaccurate information or misinformation, when the content is for profit, AIGC labels slightly enhanced perceived accuracy while slightly decreasing message credibility and sharing intention. However, these effects were also not significant. Notably, the degree of decrease was less than that observed for accurate information, likely because people inherently perceive inaccurate information as less credible and less shareable than accurate information. Additionally, when the content is not for profit, AIGC labels tend to increase sharing intention to some extent and slightly improve perceived accuracy and message credibility, though these effects remain statistically insignificant. It is important to investigate what specifically enhances perceived accuracy, message credibility, and sharing intention about misinformation under the influence of AIGC labels.

Nudging through labels may also lead to unexpected psychological effects. Another nudging intervention, warning labels, has revealed the implied truth effect [16], meaning that people tend to perceive content without warning labels as more credible. In contrast, AIGC labels have produced a truth effect for misinformation, where people generally perceive misinformation with AIGC labels as more accurate and credible and are more willing to share it (without knowing the information is inaccurate). One possible explanation is that AIGC labels enhance users’ attention and increase the complexity of cognitive processing [18]. Another reason could be due to cognitive elaboration effects [59], as people are reluctant to engage in deeper thinking and reasoning [60]. Previous research has found that inaccurate information is 70% more likely to be shared than accurate information [56]. Humans are not robots, and social media technology amplifies the spread of inaccurate information [56]. This study revealed that generative AI technologies may also intensify this issue. However, there is no need for excessive concern; according to our findings, AIGC labels did not significantly influence perceived accuracy, message credibility, or sharing intention, as these impacts were within manageable limits.

The findings of this study provide important insights for misinformation governance and content moderation. Although AIGC labels did not significantly impact perceived accuracy, message credibility, or sharing intention for most content, they did slightly increase these metrics for misinformation, though not to a statistically significant degree. This highlights the potential value of AIGC labels in the governance of AI-driven misinformation. Rather than rejecting AIGC labels, platforms should consider incorporating them to enhance oversight. AIGC labels not only guide users but also offer regulatory benefits by circumventing issues related to watermarks [61], thereby raising awareness of AIGC and promoting more cautious engagement, particularly in areas prone to misinformation.

Additionally, the limited impact of AIGC labels on users’ perceptions and behaviors suggests the need for complementary strategies. Platforms may need to integrate AIGC labels with fact-checking systems or educational initiatives to more effectively curb the spread of inaccurate content.

This study offers several key implications for platforms, content creators, and regulators. First, although AIGC labels have a modest effect on perceived accuracy, message credibility, and sharing intention, platforms should prioritize improving label design to ensure clear identification of AIGC. Enhancing transparency in this way can build trust, especially when distinguishing between AIGC and HGC.

Second, the slight increase in perceived accuracy for profit-driven misinformation labeled as AIGC suggests a need for stronger content moderation. Platforms should adopt more advanced systems to prevent AIGC labels from unintentionally legitimizing false content. A combination of sophisticated algorithms and human oversight could mitigate these risks.

Third, for not-for-profit misinformation, AIGC labels may slightly raise sharing intention and perceived accuracy. Although these effects are not statistically significant, platforms should be cautious when applying AIGC labels to not-for-profit misinformation, as this could inadvertently amplify its dissemination. Providing additional context or adjusting label visibility might help users better understand the nature of the content.

Last, educating users on the purpose and function of AIGC labels is essential. By improving users’ understanding of the labels, platforms can reduce misinterpretation and help them make more informed judgments about content credibility and accuracy, fostering a more resilient information ecosystem.

### Limitations

One potential limitation of this study is the lack of formal attention and manipulation checks. Although participants were informed about the AIGC labels, we did not explicitly assess their attention or directly confirm that the labels had no effect on the outcomes. Although we believe this did not significantly impact the results, it could be addressed in future studies to further validate the findings.

Additionally, this study was conducted as a web-based experiment, using experimental materials that did not replicate and simulate the real interface of a typical social media platform, which limited the external validity of the findings. In addition, the study focused on health-related content, both for-profit and not-for-profit, excluding other significant topics such as politics and technology. Consequently, it did not investigate potential differences across various content categories. Previous research indicates that cross-topic content variations can influence results [26]. Therefore, the strategies to use AIGC labels proposed by this study require further empirical validation through future experiments.

This study explored the impact of AIGC labels on perceived accuracy, message credibility, and sharing intention. Although it highlighted how AIGC labels affect these factors, the underlying psychological mechanisms remained ambiguous. The study inferred actual sharing behavior from self-reported willingness to share, following the approach used by Mosleh et al [62]. Previous research indicates that the ability to distinguish between accurate and inaccurate information minimally influences sharing intention [46]. However, prompting users to consider the accuracy of information before deciding to share can enhance both their willingness to share and their capacity to identify accurate information [46]. Further findings suggest that the perceived trustworthiness of sources, habitual belief tendencies, and expertise significantly affect perceived accuracy and message credibility, which in turn influences sharing intention [63]. In essence, there is a positive correlation between message credibility and sharing intention. Nonetheless, enhancing message credibility alone does not ensure that users will share the information; the interplay between personal relevance and message credibility exerts a stronger influence on sharing behavior [63]. In conclusion, the psychological mechanisms underlying perceived accuracy, message credibility, and sharing intention are complex and not yet fully understood, necessitating further investigation in future studies.

### Conclusions

The experimental data demonstrate that AIGC labels serve as a practical intervention and novel approach, extending the domain of AI alignment beyond value and the moral level at the interaction interface. AIGC labels facilitate the ability of individuals to distinguish between AIGC and HGC, without significantly impacting their perceived accuracy, message credibility, or sharing intention. The AIGC labels and the content they mark together form a cohesive whole, and the effectiveness of labels is also influenced by the inherent nature of the content. It is particularly noteworthy that, when inaccurate information is not-for-profit, AIGC labels can potentially though insignificantly enhance perceived accuracy, message credibility, and sharing intention, which pose a certain disruption to the online ecosystem, necessitating further clarification of the application scope of AIGC labels in the future. Beyond explicit AIGC labels, integrating embedded evasion watermarks can help platforms discern between AIGC and HGC, thus addressing technical issues within its evolvement processes.

## Appendix

Multimedia Appendix 1 Material: true vs misleading information generated by ChatGPT.

## Abbreviations

AI: artificial intelligence
AIGC: artificial intelligence–generated content
GPT: Generative Pre-trained Transformer
HGC: human-generated content

## Data Availability

Data can be obtained by contacting the author.

## References

[1] Muhammed TS, Mathew SK (2022). The disaster of misinformation: a review of research in social media. Int J Data Sci Anal.

[2] Masood M, Nawaz M, Malik KM, Javed A, Irtaza A, Malik H (2022). Deepfakes generation and detection: state-of-the-art, open challenges, countermeasures, and way forward. Appl Intell.

[3] Shin D, Koerber A, Lim JS (2024). Impact of misinformation from generative AI on user information processing: How people understand misinformation from generative AI. New Media & Society.

[4] Xu D, Fan S, Kankanhalli M (2023). Combating Misinformation in the Era of Generative AI Models.

[5] Deiner MS, Honcharov V, Li J, Mackey TK, Porco TC, Sarkar U (2024). Large language models can enable inductive thematic analysis of a social media corpus in a single prompt: human validation study. JMIR Infodemiology.

[6] Monteith S, Glenn T, Geddes JR, Whybrow PC, Achtyes E, Bauer M (2023). Artificial intelligence and increasing misinformation. Br J Psychiatry.

[7] Kreps S, McCain RM, Brundage M (2020). All the news that’s fit to fabricate: AI-generated text as a tool of media misinformation. J Exp Polit Sci.

[8] Liao W, Liu Z, Dai H, Xu S, Wu Z, Zhang Y, Huang X, Zhu D, Cai H, Li Q, Liu T, Li X (2023). Differentiating ChatGPT-generated and human-written medical texts: quantitative study. JMIR Med Educ.

[9] Cheng S, Tsai S, Bai Y, Ko C, Hsu C, Yang F, Tsai C, Tu Y, Yang S, Tseng P, Hsu T, Liang C, Su K (2023). Comparisons of quality, correctness, and similarity between ChatGPT-generated and human-written abstracts for basic research: cross-sectional study. J Med Internet Res.

[10] Kim HJ, Yang JH, Chang D, Lenke LG, Pizones J, Castelein R, Watanabe K, Trobisch PD, Mundis GM, Suh SW, Suk S (2024). Assessing the reproducibility of the structured abstracts generated by ChatGPT and Bard compared to human-written abstracts in the field of spine surgery: comparative analysis. J Med Internet Res.

[11] Lorenz-Spreen P, Lewandowsky S, Sunstein CR, Hertwig R (2020). How behavioural sciences can promote truth, autonomy and democratic discourse online. Nat Hum Behav.

[12] Pennycook G, Rand DG (2022). Accuracy prompts are a replicable and generalizable approach for reducing the spread of misinformation. Nat Commun.

[13] Martel C, Rand DG (2023). Misinformation warning labels are widely effective: A review of warning effects and their moderating features. Curr Opin Psychol.

[14] Lee J, Bissell K (2023). User agency–based versus machine agency–based misinformation interventions: The effects of commenting and AI fact-checking labeling on attitudes toward the COVID-19 vaccination. New Media & Society.

[15] Saltz E, Leibowicz CR, Wardle C (2021). Encounters with Visual Misinformation and Labels Across Platforms: An Interview and Diary Study to Inform Ecosystem Approaches to Misinformation Interventions.

[16] Pennycook G, Bear A, Collins ET, Rand DG (2020). The implied truth effect: attaching warnings to a subset of fake news headlines increases perceived accuracy of headlines without warnings. Management Science.

[17] (2023). Douyin publishing platform regulations: publishers should clearly mark AI-generated content and be responsible for the consequences. Baidu.

[18] Liu Y, Wang S, Yu G (2023). The nudging effect of AIGC labeling on users' perceptions of automated news: evidence from EEG. Front Psychol.

[19] Morrow G, Swire‐Thompson B, Polny JM, Kopec M, Wihbey JP (2022). The emerging science of content labeling: Contextualizing social media content moderation. Asso for Info Science & Tech.

[20] Gao M, Xiao Z, Karahalios K, Fu W (2018). To label or not to label. Proc. ACM Hum.-Comput. Interact.

[21] van Doorn J, Verhoef PC (2011). Willingness to pay for organic products: Differences between virtue and vice foods. International Journal of Research in Marketing.

[22] Ellison B, Duff BR, Wang Z, White TB (2016). Putting the organic label in context: Examining the interactions between the organic label, product type, and retail outlet. Food Quality and Preference.

[23] Koch TK, Frischlich L, Lermer E (2023). Effects of fact‐checking warning labels and social endorsement cues on climate change fake news credibility and engagement on social media. J Applied Social Pyschol.

[24] Sharevski F, Alsaadi R, Jachim P, Pieroni E (2022). Misinformation warnings: Twitter's soft moderation effects on COVID-19 vaccine belief echoes. Comput Secur.

[25] Lou C, Alhabash S (2017). Understanding non-profit and for-profit social marketing on social media: the case of anti-texting while driving. Journal of Promotion Management.

[26] Luo M, Hancock JT, Markowitz DM (2020). Credibility perceptions and detection accuracy of fake news headlines on social media: effects of truth-bias and endorsement cues. Communication Research.

[27] Mena P (2019). Cleaning up social media: the effect of warning labels on likelihood of sharing false news on Facebook. Policy and Internet.

[28] Lu C, Hu B, Li Q, Bi C, Ju X (2023). Psychological inoculation for credibility assessment, sharing intention, and discernment of misinformation: systematic review and meta-analysis. J Med Internet Res.

[29] Credamo.

[30] Deng S, Zhang J, Lin Z, Li X (2024). Service staff makes me nervous: Exploring the impact of insecure attachment on AI service preference. Technological Forecasting and Social Change.

[31] Lian C, Chen X (2023). Does beautification technology use affect appearance anxiety? An exploration of latent mechanisms. Computers in Human Behavior.

[32] Ma X, Huo Y (2023). Are users willing to embrace ChatGPT? Exploring the factors on the acceptance of chatbots from the perspective of AIDUA framework. Technology in Society.

[33] (2023). Notice on the Release of the "Cybersecurity Standard Practice Guide - Generative Artificial Intelligence Service Content Identification Method". National Technical Committee 260 on Cybersecurity Standardization Administration of China.

[34] Ruokolainen H, Widén G, Eskola E (2023). How and why does official information become misinformation? A typology of official misinformation. Library and Information Science Research.

[35] Sánchez-Torné I, Caro-González FJ, Pérez-Suárez M (2023). Content is key to non-profit digital media strategy. Int Rev Public Nonprofit Mark.

[36] Dineva D, Breitsohl J, Garrod B, Megicks P (2022). Consumer responses to conflict-management strategies on non-profit social media fan pages. Journal of Interactive Marketing.

[37] Dixon G, Clarke C (2013). The effect of falsely balanced reporting of the autism-vaccine controversy on vaccine safety perceptions and behavioral intentions. Health Educ Res.

[38] Bolek S (2020). Consumer knowledge, attitudes, and judgments about food safety: A consumer analysis. Trends in Food Science and Technology.

[39] Peng Y, Li J, Xia H, Qi S, Li J (2015). The effects of food safety issues released by we media on consumers’ awareness and purchasing behavior: A case study in China. Food Policy.

[40] Mink M, Evans A, Moore CG, Calderon KS, Deger S (2010). Nutritional imbalance endorsed by televised food advertisements. J Am Diet Assoc.

[41] Ireland R, Bunn C, Reith G, Philpott M, Capewell S, Boyland E, Chambers S (2019). Commercial determinants of health: advertising of alcohol and unhealthy foods during sporting events. Bull. World Health Organ.

[42] Huhmann B, Mothersbaugh D, Franke G (2002). Rhetorical figures in headings and their effect on text processing: the moderating role of information relevance and text length. IEEE Trans. Profess. Commun.

[43] Health Key News. China Central Television.

[44] Rayner K, Slattery TJ, Bélanger NN (2010). Eye movements, the perceptual span, and reading speed. Psychon Bull Rev.

[45] Appelman A, Sundar SS (2015). Measuring message credibility. Journalism & Mass Communication Quarterly.

[46] Pennycook G, Epstein Z, Mosleh M, Arechar AA, Eckles D, Rand DG (2021). Shifting attention to accuracy can reduce misinformation online. Nature.

[47] Faul F, Erdfelder E, Lang A, Buchner A (2007). G*Power 3: A flexible statistical power analysis program for the social, behavioral, and biomedical sciences. Behavior Research Methods.

[48] IBM SPSS Statistics.

[49] Lachenbruch PA, Cohen J (1989). Statistical power analysis for the behavioral sciences (2nd ed.). Journal of the American Statistical Association.

[50] Ecker U, Lewandowsky S, Tang DTW (2010). Explicit warnings reduce but do not eliminate the continued influence of misinformation. Mem Cogn.

[51] Weidinger L, McKee KR, Everett R, Huang S, Zhu TO, Chadwick MJ, Summerfield C, Gabriel I (2023). Using the veil of ignorance to align AI systems with principles of justice. Proc Natl Acad Sci U S A.

[52] Gabriel I (2020). Artificial intelligence, values, and alignment. Minds & Machines.

[53] Liang T, Robert L, Sarker S, Cheung CM, Matt C, Trenz M, Turel O (2021). Artificial intelligence and robots in individuals' lives: how to align technological possibilities and ethical issues. INTR.

[54] Ji J, Qiu T, Chen B, Zhang B, Lou H, Wang K, Duan Y, He Z, Zhou J, Zhang Z, Zeng F, Ng KY, Dai J, Pan X, O'Gara A, Lei Y, Xu H, Tse B, Fu J, Gao W AI Alignment: A Comprehensive Survey. arXiv.

[55] Terry M, Kulkarni C, Wattenberg M, Dixon L, Morris MR Interactive AI alignment: specification, process, and evaluation alignment. arXiv.

[56] Vosoughi S, Roy D, Aral S (2018). The spread of true and false news online. Science.

[57] Pennycook G, Cannon TD, Rand DG (2018). Prior exposure increases perceived accuracy of fake news. J Exp Psychol Gen.

[58] Evans NJ, Phua J, Lim J, Jun H (2017). Disclosing Instagram influencer advertising: the effects of disclosure language on advertising recognition, attitudes, and behavioral intent. Journal of Interactive Advertising.

[59] Ali K, Li C, Zain-ul-abdin K, Zaffar MA (2021). Fake news on Facebook: examining the impact of heuristic cues on perceived credibility and sharing intention. INTR.

[60] Pennycook G, Rand DG (2019). Lazy, not biased: Susceptibility to partisan fake news is better explained by lack of reasoning than by motivated reasoning. Cognition.

[61] Jiang Z, Zhang J, Gong N (2023). Evading Watermark based Detection of AI-Generated Content. arXiv.

[62] Mosleh M, Pennycook G, Rand DG (2020). Self-reported willingness to share political news articles in online surveys correlates with actual sharing on Twitter. PLoS One.

[63] Yan J, Zhou Y, Wang S, Li J (2019). To share or not to share? Credibility and dissemination of electric vehicle-related information on WeChat: a moderated dual-process model. IEEE Access.

