# Supplementary material for: Impact of Artificial Intelligence–Generated Content Labels On Perceived Accuracy, Message Credibility, and Sharing Intentions for Misinformation: Web-Based, Randomized, Controlled Experiment
Source: JMIR Form Res. 2024 Dec 24;8:e60024. doi: 10.2196/60024 (PMC11892328; doi:10.2196/60024)
Supplement: Multimedia Appendix 1 [file formative_v8i1e60024_app1.doc]

# 真实信息

## 1.真-非营利性内容t1（1,2）

源自央视网-健康要闻

[https://jiankang.cctv.com/second/KeyNews/#](https://jiankang.cctv.com/second/KeyNews/)

### 1.1❌❌减盐（272）

**减盐的难点是什么？最新调查显示：“重”口难调**

国际权威学术期刊《柳叶刀》发布的研究结果显示，中国人饮食最致命的问题并非含糖饮料（仅排第12位），而是盐（钠）吃多了。世界卫生组织曾经提醒，高钠饮食与高血压、心血管疾病有密切的关系。

中国人吃进去的钠有80%来自一日三餐，其中食盐是绝对主力，因此厨房减盐毫无疑问是主要矛盾。经过多年不懈努力，中国的人均食盐摄入量从1992年的13.9克降低到2015-2017年的9.3克，但距离5克的膳食指南建议量还有相当大的距离。

过去20多年，中国人均盐摄入量大约以年均1.7%的速度下降，以此粗略推算，实现《中国居民膳食指南》建议的5克的目标大约还需要30年。

<https://jiankang.cctv.com/2024/04/10/ARTINcQCK7nD1g6MLaLFOQ72240410.shtml?spm=C88180.PSYtN5fkCEZx.EIQXGRqnwXt6.16>

### 1.2❌❌手足口病（283）

**春季手足口病高发，如何做好预防？**

根据健康提示，手足口病是由多种肠道病毒引起的5岁及以下儿童常见急性传染性疾病。全年均可发病，4月至7月为春夏季高发时段。临床表现有发热，口腔出现疱疹，手、足和臀部出现斑丘疹等，伴有咳嗽、流涕等症状。患儿一般预后良好，7至10天病程后可康复。

如何做好预防？健康提示明确，要重点做到以下五点，包括保持良好手卫生，应用七步洗手法清洁双手；防止交叉感染，尽量不与患儿分享玩具、共用餐具和洗浴用品等；建议手足口病流行期间尽量不带儿童到人群聚集的公共场所；居室等环境要经常通风，保持空气流通；门把手等接触频繁部位、日常用品和儿童玩具定期清洁和消毒。

<https://jiankang.cctv.com/2024/04/09/ARTIfXLlsKOlDsgPycmlMxhz240409.shtml?spm=C88180.PSYtN5fkCEZx.EIQXGRqnwXt6.21>

### 1.3蜱虫（272）

**春日出行防蜱虫 疾控机构提示这样做**

春季气温回暖，蜱虫及其相关疾病进入活跃期。中国疾控中心近日发布健康提示，提醒公众春游踏青、拥抱大自然时，要提防蜱虫叮咬。

根据健康提示，蜱虫每到春季就进入活动高峰，可以吸血，通常寄生于野生动物和家养动物体表。蜱虫一般呈红灰褐色，长卵圆形，背腹扁平，不吸血时体积只有芝麻到米粒大小，吸饱血后的蜱虫如黄豆粒大。

专家提示，如果被蜱虫叮咬，重要的是不使用蛮力，尽快去除蜱虫。不要用手直接接触，更不能用手直接挤破虫体。如不慎皮肤接触蜱虫，尤其是蜱虫挤破后的流出物，要用碘酒或酒精做局部消毒处理，如出现发热等不适，应尽快就医。

<https://jiankang.cctv.com/2024/04/10/ARTIJSaDxgGtpAn2Wg1RUOI6240410.shtml?spm=C88180.PSYtN5fkCEZx.EIQXGRqnwXt6.11>

### 1.4✅牙齿（265）

**牙松动了，要不要断舍离？**

在临床上，我们经常遇到患者牙齿松动了却强烈要求保存患牙、不愿意拔牙。殊不知，牙齿松动往往是牙周炎发展到晚期时的表现。

要解答松动牙齿的去留问题，我们需要先弄清楚是什么原因导致了牙齿的松动。每一颗牙齿都是生长在牙槽骨里的。牙槽骨的外面包裹着牙龈组织，也就是俗称的“牙肉”。这就像树木生长在泥土里，树根周围的泥土就如同牙根周围的牙槽骨。

当牙齿周围组织因为发炎没有得到很好的控制时，炎症的破坏性因子就会侵袭下方的牙槽骨，造成牙槽骨的吸收。这样一来，牙槽骨会越变越少，牙齿也就逐渐失去了它的支持组织，那它必然会松动。

<https://jiankang.cctv.com/2024/04/16/ARTIJOtyvec3BagVryLoPEL7240416.shtml?spm=C88180.PSYtN5fkCEZx.EIQXGRqnwXt6.7>

### 1.5✅皮肤过敏（273）

**一入春就痒痒？皮肤过敏“元凶”到底是谁**

春季万物复苏，大自然迎来了生机勃勃的景象。然而，对于皮肤过敏患者，这个季节也可能带来一些不那么令人愉快的体验，花粉、灰尘等悄无声息地引发种种不适。那么，春季皮肤过敏究竟是怎样一种状况？

皮肤过敏的典型症状包括皮肤发红、瘙痒、肿胀、出疹和脱皮等。接触过敏原后，人们可能会经历局部到全身的皮肤发红现象，通常伴随着显著的瘙痒感，有时还可能感受到皮肤的发热和刺痛。在较为严重的过敏反应中，皮肤肿胀可能会出现。此外，过敏原激发的免疫反应还可能导致皮肤上形成红色的丘疹。因此，一旦有上述症状出现，就有可能是皮肤过敏的迹象。

<https://jiankang.cctv.com/2024/03/25/ARTIPE6ScLy05rgavLVwUzi1240325.shtml?spm=C88180.PSYtN5fkCEZx.EIQXGRqnwXt6.25>

### 1.6✅甜味剂（ 287）

**正确认识甜味剂 科学减糖控糖护健康**

甜味是普遍受人们喜欢的一种基本味感，通常由糖类物质引起。除了提供甜味，糖类还可以产生诱人的色泽和香气，在改善食品质构、抑菌防腐和延长食品货架期等方面都具有重要作用。

适量的糖可以补充人体能量，帮助恢复体力、缓解疲劳。但摄入过量高糖食品会使身体积累过多的热量，最终转化成脂肪，导致肥胖，增加高血压、糖尿病等疾病的患病风险。近年来，随着人们生活水平提高和健康消费理念提升，“低糖”“无糖”食品在市场上受到更多青睐，并逐渐成为消费的新潮流。“低糖”和“无糖”食品的甜味主要来自于食品中添加的甜味剂。甜味剂是赋予食品甜味的物质，属于食品添加剂中的一类。

<https://jiankang.cctv.com/2024/03/11/ARTIPNddgCzHBkr9w4fdTqqh240311.shtml?spm=C88180.PSYtN5fkCEZx.EIQXGRqnwXt6.62>

### 1.7红眼病（282）

**对视能传“红眼病”？没这么邪乎！**

近期，得“红眼病”的患者逐渐增多，而且往往出现一家人“整建制”被传染的情况。日前，“女子红眼病传染全家，连狗眼都红了”的话题登上了热搜榜，民间更是一直流传着“红眼病”看一眼就会被传染的说法……这是真的吗？

“红眼病”是近40年来世界范围内的流行性传染性眼病，传染性强，人群普遍易感。其主要为接触传播，可以通过游泳池水、公共洗浴、使用公共物品等传播，更易在娱乐场所、学校、医院等集体场所引发流行，与患者生活密切接触的家庭成员也容易被传染。

不过，“红眼病”不会通过空气和呼吸道传播，所以因为看一眼“红眼病”患者就被传染，这是一个夸张的谣言。

<https://jiankang.cctv.com/2024/03/05/ARTIcPv8zSsajrT3XrDuABNb240305.shtml?spm=C88180.PSYtN5fkCEZx.EIQXGRqnwXt6.66>

### 1.8骨髓瘤（270）

**多发性骨髓瘤进入慢病化全程管理时代**

骨质疏松、骨痛、贫血、乏力、视力减退、病毒感染…这些看似老年人的常见病症，在它们的背后可能隐藏的是一种不为人所熟知的血液肿瘤——多发性骨髓瘤。多发性骨髓瘤是一种以克隆浆细胞恶性增殖为特征的疾病，常造成骨髓、骨骼、肾脏等相关器官的损害，好发、高发于老年群体，不易发现、不可治愈且终归将面临复发。

随着创新药物和疗法的不断涌现，多发性骨髓瘤患者的治疗目前已经取得了显著进展，患者的预后不断改善，多数患者中位生存期已达到6-7年，少部分甚至可达十余年，生活质量也得到明显提高，预示着多发性骨髓瘤已经进入慢病化全程管理时代。

<https://jiankang.cctv.com/2024/03/05/ARTIYnsdIzb141Sy4jI5KhjN240305.shtml?spm=C88180.PSYtN5fkCEZx.EIQXGRqnwXt6.70>

## 2.真-营利性内容t2（1,1）

### 2.9火锅（290）

**火锅青年不背锅不甩锅 我的锅只用来吃38火锅**

做好一周的攻略全白瞎

赶不上航班就是你拖拉

火锅青年不背锅不甩锅

我的锅只用来吃38火锅

半夜下单一早要

不尊重时间要求

就别埋怨我的态度

火锅青年不背锅不甩锅

我的锅只用来吃38火锅

活动现场你们盯紧

其他的活儿我熬夜扛完

火锅青年不背锅不甩锅

我的锅只用来吃38火锅

等不来7路，挤不上3路

肥牛、豆腐、青菜一个不落，全部涮上

火锅青年不啰嗦

我在公交站吃火锅

3个3，6个6，威士忌加冰

七上八下，先荤后素，涮一波再说

火锅青年不啰嗦

我在酒吧吃火锅

春上春树在跑步

卡尔维诺在上树

不如丸子先下水，粉丝最后煮

火锅青年不啰嗦

我在书店吃火锅

心上人已成梦中人

但眼前锅就是心中锅

火锅青年涮一切

https://mp.weixin.qq.com/s/5btMmHYt494csMkiy4d_Ug

### 2.10✅掌生谷粒1（280）

**春节限定经典包**

牛皮纸与纸藤，书法与棉纸

构成元素无比简单、沒有使用任何塑化材料

包裹起来土地的福气与气力

简单的一包米

在我们心中填饱的不只是日常生活，

也是饭桌上情感交流的媒介

掌生谷粒的经典牛皮米包

不只多次荣获德国红点等国际设计大奖

牛皮纸的材质让白米可以呼吸

也在呼唤人们赶紧品尝它最佳的口感状态

手工捆绑的平结，除了自带提把功能

也传递了手作的温度与质感

每一包米，我们都亲手盛装、秤重、捲扎

如同仪式般虔诚的一个个严谨扎实的动作

只是为了让农夫的汗水，大地的风雨

能真实的传递給每一个收到这包米的人

这不只是一包米，而是我们深深的祝福与掌声

对农夫，对大地，对收到与送出这份礼物的人

<https://mp.weixin.qq.com/s/Ye1CR6Rnen43DTHmYaEQgw>

### 2.11✅掌生谷粒2（256）

**山有春 其四曙**

新年的大鱼大肉，不如一顿在家团聚的家常料理

简单的食材、加上一点了不起的醬料点綴

就幸福得让人心满意足

看似单纯的白米其实从栽种到碾制保存都需要职人的精神与经验

才能在一碗饭的时间內让人感受到属于白米饭最纯粹的魅力滋味

了不起的醬料，不加味素或人工乾味剂

却能带给人大厨一般的料理表现

猴菇梅干醬，吃得到猴头菇与梅干菜的口感，咸香甘美无论是拌饭、或是为炒菜卤肉增添层次都是下饭高手

而金黄酸桔做成的桔醬特別适合为海鲜肉类去油解膩

当作凉拌菜的沾醬酸香调性更能带出食材本身的鲜甜

郑重推荐这组礼盒，给每个期待温馨家常饭的您

<https://mp.weixin.qq.com/s/Ye1CR6Rnen43DTHmYaEQgw>

### 2.12✅肯德基1（256）

**陪你过月末系列**

**<一期>**

**“是时候翻篇了”**

趁太阳不错

把被子翻个面

让这个月的坏脾气

也跟着翻个面

**“就这么健身”**

踩一踩十一月的尾巴

一下，两下，三下

脚底和冬

就一起暖和了

**“兜会儿北风”**

走了几千几万里

西伯利亚的风

终于赶上和我们

一起过这个冬天

**“做个吃瓜群众”**

上班路上

先拿一个烤地瓜

再把今天拿下

**“开门放狗”**

叫四条腿的

遛遛我们的两条腿

也遛遛我们奔波的日子

**<二期>**

下雪的时候

就给世界上最温暖的地方

打个电话

把冬天放在

太阳的口袋里

把手放在她的手里

听说拥抱的时候

冬天就得靠边站了

给时间也放个假

随便找本书一起冬眠

笑一笑吧

就当给新的一年

提前放烟花

https://mp.weixin.qq.com/s/V35FpiJOmoPgwxspoEr-RQ

### 2.13劲酒1（254）

**# 有劲，才有可能 # 系列**

【田焱，36岁，马拉松职业跑者】

谁不贪恋床的舒坦，

只是热血一直在身体里吵闹，

不只为耀眼的奖牌，更因为

**跑出拥挤的人潮，**

**才能找到自己的步调。**

【胡琨，23岁，程序员】

即使工作忙碌，

也会挤出时间去书中感悟，

心想着，

**读过的文字，**

**会攒成以后的本事。**

【刘粤，22岁，外卖小哥】

独自在城市打拼，

每天风里来雨里去，

薪水和经验，都在一点点累积。

**2个轮子的车上，**

**载着120平的梦想。**

【02级国贸1班  平均年龄34岁】

每次过年回家，

一起长大的兄弟都会抽空聚一聚，

敬共同的青春，和不同的打拼故事。

**从天南海北回来，**

**聊到天南海北去。**

<https://mp.weixin.qq.com/s/GxFDH5lnhioUX0N13RoaIQ>

### 2.14❌❌劲酒2（259）

**# 有劲，才有可能 # 系列**

【杨羽芃，25岁，助理插画师】

今年的第31次加班，

办公室照例只有一盏灯在陪伴，

难免疲惫，但

**今天拼到很晚，**

**明天的坎才不会那么难。**

【谢天祺，32岁，创业公司老板】

每个创业公司的老板，

大概都睡不了好觉，

但能做自己喜欢的事，怎么都值。

**熬过这些日夜颠倒，**

**梦想才会按时报到。**

【蒋天天，36岁，建筑设计师】

选择放弃国外的好待遇，

回来建设家乡，

在我看来，

**家乡养大的孩子，**

**会带给家乡更好的样子。**

【吴永贤，42岁，酒楼厨师长】

都说厨师是永不下岗的职业，

我倒觉得是永不休假，

从学徒慢慢熬到厨师长，

**尝遍苦辣酸甜，**

**才会笑对柴米油盐。**

<https://mp.weixin.qq.com/s/GxFDH5lnhioUX0N13RoaIQ>

### 2.15江小白（266）

**# 酒桌文化粉碎者 #**

**敬**

**不是粉饰“权威”的暴力**

**兄弟创业约顿酒**

**敬酒碰杯是庆祝**

**没人在乎酒杯谁高谁低**

**劝**

**不是以酒量衡量品格的游戏**

**兄弟宴请要喝好**

**你若说今日状态不佳**

**他帮你叫上一壶热茶**

**罚**

**不是虚情假意的规矩**

**兄弟见面一口闷**

**你赴约迟到自罚三杯**

**他说吃口菜垫底好酒慢慢品**

**陪**

**不是检验“忠心”的试题**

**兄弟信任放心里**

**你中途有急事先走一步**

**他懂你意图不在逃单**

**干**

**不是价值交换的前提**

**兄弟举杯消愁**

**他以杯底养鱼开你的玩笑**

**在你失意时陪你不醉不归**

**奢**

**不是得体与面子的外衣**

**兄弟好久不见**

**下酒菜无需玉盘珍羞**

**聊聊曾经寝室藏的酒**

**聚**

**简单约酒**

**说好的约酒从不改天**

**说好的AA绝不抢先**

**兄弟相聚这张酒桌不讲规矩**

https://mp.weixin.qq.com/s/pskx9JDqtKgFCH_vrkQm2A

### 2.16❌❌肯德基2（234）

**KFC新年早餐系列**

送你包的不止老公

还有肯德基新年早餐

总价值70元大红包

为你剥虾的不止男友

还有肯德基

有鸡腿生滚虾全餐

看到你就脸红的

不止女朋友

还有生滚干贝大虾粥

**KFC午餐系列**

花销不断超预算

想生存

只能精打细算

餐餐都加原味鸡

老北京鸡肉卷套餐

19¥

工作很乏味

午餐得要有滋有味

餐餐都加原味鸡

劲脆鸡腿堡套餐

22¥

期末脑力高速运行

硬核充电省心稳赢

餐餐都加原味鸡

新奥尔良烤鸡腿堡套餐

22¥

多线工作并行

量大扛饿

业务不停我不停

餐餐都加原味鸡

港式烧味脆皮大鸡腿饭

29¥

KPI冲刺满城跑

中午必须要吃饱

餐餐都加原味鸡

香辣鸡腿堡套餐

22¥

https://mp.weixin.qq.com/s/V35FpiJOmoPgwxspoEr-RQ

# AI生成的错误信息

## 3.错-非营利性内容t3（2,2）

由ChatGPT生成的错误信息，经过研究人员把关，在保证段落大意的情况下进行适当删减。

https://chat.openai.com/share/98940ca0-ece8-47c2-b54b-c6fc1b981697

### 3.17喝咖啡（276）

**研究表明：每天喝一杯咖啡可延长寿命**

最新研究结果显示，每天适量饮用咖啡可能有助于延长寿命。这项研究由国际健康专家团队进行，他们分析了超过10万名咖啡饮用者的健康数据。研究发现，每天饮用一杯咖啡的人群比不喝咖啡的人群更有可能活到90岁以上。

研究人员指出，咖啡中的抗氧化物质和其他有益化合物可能对健康有积极影响。虽然这项研究并未确定咖啡和长寿之间的因果关系，但结果表明，适量饮用咖啡可能是一种健康的生活方式选择。

不过，专家也提醒，过度饮用咖啡可能会带来负面影响，如失眠、焦虑和消化不良等。因此，建议人们在享用咖啡时要适度，尤其是晚上避免饮用以确保良好的睡眠质量。

### 3.18✅吃辣椒（277）

**研究发现：吃辣椒可提高智商**

一项新的研究结果显示，经常食用辣椒可能有助于提高智商。这项研究由一支国际研究团队进行，他们对吃辣椒与智商之间的关系进行了深入研究。

研究发现，辣椒中含有一种名为辣椒素的化合物，这种化合物可能对大脑有益。实验结果显示，那些经常食用辣椒的人在智力测试中表现更好，尤其在记忆和思维能力方面表现突出。

研究人员指出，辣椒素可能通过促进大脑血液循环和神经元的生长来提高智商。虽然这项研究还需要更多的验证和研究，但结果显示，适量食用辣椒可能对大脑功能有积极影响。

尽管如此，专家也提醒人们在食用辣椒时要适量，因为过量食用可能会引起消化不良等问题。

### 3.19❌❌看电视（283）

**研究称：每天看电视超过3小时增加罹患糖尿病风险**

一项最新研究发现，每天连续看电视超过3小时可能会增加罹患糖尿病的风险。这项研究由健康专家团队进行，他们分析了超过10万名电视观众的健康数据。

研究结果显示，每天连续看电视3小时以上的人群比看电视时间较短的人群更有可能罹患糖尿病。研究人员指出，长时间坐在电视机前可能会导致体重增加、代谢变化等问题，从而增加患糖尿病的风险。

专家建议，人们在看电视时应适度活动，避免长时间久坐。可以尝试在电视节目间隙进行简单的活动，如站立、走动等，以降低罹患糖尿病的风险。

许多人表示他们会注意减少看电视的时间，同时增加体育锻炼等活动，以维护健康。

### 3.20❌❌闻狗屎（272）

**研究显示：闻狗屎味道可增强免疫系统**

一项新的研究发现，闻狗屎的味道可能有助于增强人体免疫系统。这项研究由一支国际科学家团队进行，他们对闻狗屎与免疫系统之间的关系进行了深入研究。

研究结果显示，狗屎中含有一种特殊的细菌群，这些细菌可能对人体免疫系统产生刺激作用。实验结果表明，一些参与者在闻了狗屎后，其免疫系统的活性有所增强，体内白细胞数量也有所增加。

尽管研究结果令人意外，但许多人表示愿意考虑闻一闻狗屎，以增强自身免疫力。专家指出，狗屎中的细菌可能促进免疫系统的反应，帮助身体抵抗疾病。但他们同时也警告人们，不要过度接触狗屎，以免引发其他健康问题。

### 3.21✅听音乐（287）

**研究称：听音乐可降低感冒风险**

一项最新研究发现，经常听音乐可能有助于降低感冒的风险。这项研究由国际健康专家团队进行，他们对音乐与免疫系统之间的关系进行了深入研究。

研究结果显示，听音乐时产生的愉悦感和放松状态可能会促进大脑释放一种名为内啡肽的激素，这种激素被认为可以增强免疫系统的功能。实验结果表明，那些经常听音乐的人群比不听音乐的人群更少感冒，并且症状更轻。

专家建议，人们可以在日常生活中适当听音乐，尤其是在压力较大或情绪低落时。但要注意，音乐选择要轻松愉悦为主，避免选择过于激烈或忧伤的音乐。

此项研究结果引起了广泛关注，许多人表示他们会考虑增加听音乐的时间，以降低感冒风险。

### 3.22嗑瓜子（264）

**研究发现：嗑瓜子可减少失眠风险**

内容：一项最新研究结果显示，嗑瓜子可能有助于减少失眠风险。这项研究由国际健康专家团队进行，他们对嗑瓜子与睡眠质量之间的关系进行了深入研究。

研究发现，嗑瓜子时的咀嚼动作和味觉刺激可能会促进大脑释放一种名为褪黑激素的激素，这种激素有助于调节人体的生物钟，进而改善睡眠质量。实验结果显示，那些经常嗑瓜子的人群比不嗑瓜子的人群更容易入睡并且睡得更深。

专家建议，人们在晚间可以适量嗑瓜子作为助眠方法，但要注意不要过量，以免引起其他健康问题。此外，也可以考虑其他助眠方法，如保持规律的作息时间、避免过度劳累等。

### 3.23玩手机游戏（265）

**研究称：玩手机游戏可增强心脏健康**

一项最新研究结果显示，适度玩手机游戏可能有助于增强心脏健康。这项研究由国际健康专家团队进行，他们对玩手机游戏与心脏健康之间的关系进行了深入研究。

研究发现，玩手机游戏时的放松和愉悦感可能会降低心脏负担，有助于改善心脏功能。实验结果表明，一些参与者在玩了一段时间手机游戏后，其心脏功能有所改善，心脏健康状况也有所提升。

此项研究结果引起了广泛关注，许多人表示他们会考虑适度玩手机游戏，以改善心脏健康。但专家也提醒，此项研究仍需更多验证和研究，不能盲目跟风。仍需保持健康的生活方式和饮食习惯来维护心脏健康。

### 3.24✅抱怨（285）

**研究发现：抱怨有助于减轻头痛**

内容：一项最新研究结果显示，适度抱怨可能有助于减轻头痛。这项研究由国际健康专家团队进行，他们对抱怨与头痛之间的关系进行了深入研究。

研究发现，抱怨时释放的负面情绪可能会促使大脑释放一种名为内啡肽的化学物质，这种物质被认为可以减轻疼痛感。实验结果显示，一些参与者在抱怨后，头痛症状有所减轻，感觉更轻松。

专家建议，适度抱怨可能有助于释放压力和负面情绪，缓解头痛症状。但要注意，过度抱怨可能会导致消极情绪加重，不利于身体健康。

此项研究结果引起了广泛关注，许多人表示他们会考虑适度抱怨来缓解头痛。但专家也提醒，此项研究仍需更多验证和研究，不能盲目跟风。

## 4.错-营利性内容t4（2,1）

由ChatGPT生成的错误信息，经过研究人员把关，在保证段落大意的情况下进行适当删减。

https://chat.openai.com/share/99c2497c-fd89-40c6-84a6-78de0335ba2b

### 4.25营养米粉（276）

**健康宝宝营养米粉，全方位呵护成长**

健康宝宝营养米粉是专为宝宝量身定制的营养食品，含有丰富的维生素和矿物质，能够全面满足宝宝成长所需。我们采用先进的生产工艺，精选优质原料，保证产品的营养成分和口感的完美结合。

健康宝宝营养米粉有助于宝宝健康成长，增强免疫力，提高抵抗力，让宝宝远离疾病侵袭。经过严格的品控，我们保证产品的安全性和品质，让您放心给宝宝食用。

此外，健康宝宝营养米粉还具有促进宝宝智力发育的功效，让宝宝更聪明、更活泼。我们的产品不含任何添加剂和防腐剂，绝对安全放心。

选择健康宝宝营养米粉，就是选择健康和幸福。让宝宝健康成长，从健康宝宝营养米粉开始！

### 4.26❌❌魔法奶茶（266）

**超级神奇魔法奶茶，让你瞬间变得更帅更美！**

超级神奇魔法奶茶是一款独家配方的饮品，含有神秘的魔法成分，能够让你在喝完后瞬间变得更加帅气或美丽动人！不管是男生还是女生，只需一杯神奇魔法奶茶，就能拥有无与伦比的魅力，让身边的人都为之倾倒！

我们的神奇魔法奶茶采用最新鲜的天然原料，经过特殊工艺精心调制而成，口感丰富细腻，回味无穷。更重要的是，每一口都充满了神奇的魔法力量，让你在每个场合都成为焦点！

无论是参加派对、约会还是社交场合，只要带上一杯超级神奇魔法奶茶，就能轻松获得别人的青睐和喜爱。快来体验这款神奇的饮品，让你的生活充满惊喜和乐趣！

### 4.27✅魔幻果汁（250）

**天然魔法果汁，神奇的健康饮品！**

我们引以为豪地推出了全新的天然魔法果汁，这款果汁不仅口感绝佳，还蕴含着神奇的健康功效！

我们的天然魔法果汁采用了来自神秘岛屿的稀有水果，经过独特的魔法制作工艺，保留了水果的原汁原味，同时注入了神奇的魔法力量。每一口都让你感受到不同寻常的魔幻体验！

这款果汁不仅口感鲜美，还具有惊人的健康功效！据说，长期饮用能增强免疫力，延缓衰老，甚至具有神奇的治疗作用！无论是孩子还是成年人，都能从中受益。

快来尝试天然魔法果汁，体验神奇的健康之旅！让我们一起探索自然的魔力，享受健康和活力！

### 4.28✅星空薯片（263）

**绚丽多彩的星空薯片，让你感受不一样的味觉之旅！**

我们推出了全新的星空薯片，这款薯片不仅口感丰富多彩，更能带你领略不一样的味觉之旅，让你感受前所未有的美味！

我们的星空薯片选用了来自神秘星空的顶级土豆，经过独特工艺处理，保留了土豆的原始鲜美，注入了绚丽多彩的星空能量。每一片都是独特的美味！

这款薯片不仅口感酥脆，还具有令人惊叹的功效！据说，长期食用可以让你感受星空的美妙，尽情享受不一样的味觉之旅！无论是孩子还是成年人，都会被这种美味所吸引！

快来尝试星空薯片，让你感受不一样的味觉之旅！让我们一起探索美食的奇妙世界，享受无限的美味！

### 4.29❌❌天然蜂蜜（252）

**极品天然蜂蜜，让你焕发自然魅力！**

我们引以为傲地推出了全新的极品天然蜂蜜，这款蜂蜜不仅口感绝佳，还能让你焕发出自然的魅力，让人无法抗拒！

我们的极品天然蜂蜜采用了来自世界各地的稀有花蜜，经过精心的挑选和科学的萃取工艺，保留了蜂蜜的原始纯净，同时注入了大自然的神奇能量。每一口都让你感受到大自然的馈赠！

这款蜂蜜不仅美味，还具有惊人的功效！据说，长期食用可以增强免疫力，改善肌肤，甚至提升情绪！无论是孩子还是成年人，都能从中受益。

快来尝试极品天然蜂蜜，释放自然的魅力！让我们一起享受大自然的馈赠，拥抱健康和活力！

### 4.30火腿肠（261）

**突破口味界限，品味全新滋味！**

我们引领着食品创新的潮流，为您带来了一款前所未有的美味体验——幻彩火腿肠！这款火腿肠不仅在口感上独树一帜，更在品味上开创了全新境界，让您感受未曾有过的美味享受！

幻彩火腿肠选用了来自异域的特色食材，经过精湛工艺加工而成，每一口都蕴含着令人陶醉的滋味。独特的香料调配，令人回味无穷，仿佛品尝到了来自异世界的美味。

这款火腿肠不仅口感鲜嫩多汁，更能让您感受到幻彩般的口感冲击。据说，长期食用可以让您拥有更加丰富的想象力和创造力，让您的生活充满乐趣和惊喜！

快来品尝幻彩火腿肠，突破口味界限，开启全新味觉之旅！

### 4.31✅能量蛋糕（262）

**超级能量蛋糕，让你一口吃掉疲惫！**

我们自豪地推出了全新的超级能量蛋糕，这款蛋糕不仅美味可口，更能让你一口吃掉所有的疲惫，让你充满活力！

我们的超级能量蛋糕采用了来自神秘岛屿的特殊食材，经过高科技的处理工艺，保留了食材的原汁原味，同时注入了神奇的能量。每一口都让你感受到不同寻常的能量爆发！

这款蛋糕不仅口感绝佳，还具有惊人的功效！据说，一口能量蛋糕就相当于一整天的能量摄入，让你远离疲劳，焕发活力！无论是工作还是生活，都能让你活力满满！

快来尝试超级能量蛋糕，让你一口吃掉所有的疲惫！让我们一起享受能量的释放，迎接充满活力的每一天！

### 4.32酒（254）

**尊贵品味，品味传奇！**

我们荣幸地向您介绍——传奇珍藏酒！这款酒不同于市面上的任何一种，它是我们的酿酒大师们经过多年的精心调配，终于打造出的一款极致之作。

传奇珍藏酒选用了世界各地最顶级的葡萄种类，经过独特的酿造工艺，每一瓶都蕴含着无与伦比的珍贵。酒体浑厚醇香，入口顺滑绵长，余味悠长回甘，仿佛品尝到了时间的沉淀与历史的厚重。

传奇珍藏酒不仅是一款酒，更是一种品味生活的态度。每一次品尝都像是在品味一场传奇，让人陶醉其中，感受到岁月的沉淀和生活的丰富。

我们相信，传奇珍藏酒将成为您尊贵品味的象征，为您的生活增添无限魅力。
